# Hand-held echocardiography: added value in clinical cardiological assessment

**DOI:** 10.1186/1476-7120-3-7

**Published:** 2005-03-24

**Authors:** Giovanna Giannotti, Sergio Mondillo, Maurizio Galderisi, Riccardo Barbati, Valerio Zacà, Piercarlo Ballo, Eustachio Agricola, Francesco Guerrini

**Affiliations:** 1 Division of Cardiology, University of Siena, Italy; 2 Division of Cardiology, University Federico II, Naples, Italy; 3 Division of Cardiology, La Spezia, Italy; 4 Division of Non-Invasive Cardiology, Cardiothoracic Department, San Raffaele Hospital, IRCCS, Milano, Italy

**Keywords:** echocardiography, Hand Held Device, Standard Echocardiographic Device, conclusiveness, agreement

## Abstract

**Background:**

The ultrasonic industry has recently produced echocardiographic Hand Held Devices (miniaturized, compact and battery-equipped echocardiographic systems). Their potential usefulness has been successfully assessed in a wide range of clinical conditions. The aim of the study was to verify if the routine use of a basic model of echocardiographic Hand Held Device (HHD) could be an important diagnostic tool during outpatient cardiologic consulting or in non-cardiologic hospital sections.

**Methods:**

87 consecutive patients were included in this study; they underwent routine physical examination, resting ECG and echocardiographic evaluation using a basic model of HHD performed by trained echocardiographists; the cardiologist, whenever possible, formulated a diagnosis. The percentage of subjects in whom the findings were judged reasonably adequate for final diagnostic and therapeutic conclusions was used to quantify the "conclusiveness" of HHD evaluation. Successively, all patients underwent a second echocardiographic evaluation, by an examiner with similar echocardiographic experience, performed using a Standard Echo Device (SED). The agreement between the first and the second echocardiographic exam was also assessed.

**Results:**

Mean examination time was 6.7 ± 1.5 min. using HHD *vs. *13.6 ± 2.4 min. using SED. The echocardiographic examination performed using HHD was considered satisfactory in 74/87 patients (85.1% conclusiveness). Among the 74 patients for whom the examination was conclusive, the diagnosis was concordant with that obtained with the SED examination in 62 cases (83.8% agreement).

**Conclusion:**

HHD may generally allow a reliable cardiologic basic evaluation of outpatient or subjects admitted to non-cardiologic sections, more specifically in particular subgroups of patients, with a gain in terms of time, shortening patient waiting lists and reducing healthy costs.

## Background

Ever since the birth of echocardiography, 50 years ago, this non-invasive diagnostic tool became a milestone in the clinical evaluation of cardiovascular patients, due to its diagnostic accuracy. Owing to advances in technology, the ultrasonic industry has recently produced hand-held devices (HHD) that are miniaturized, compact and battery-equipped echocardiographic systems. Basic versions of HHD usually allow only bidimensional imaging and colour flow Doppler analysis, but M-Mode visualization, continuous and pulsed wave Doppler imaging, ECG synchronization and other tools have recently been integrated in better-equipped HHD. These machines could offer some advantages compared with standard echocardiographic devices (SED), due to their simplicity of use, immediate availability at the patient's bedside, transportability and relatively low cost.

The potential usefulness of HHD has been successfully assessed in a wide range of clinical conditions. HHD has been reported to improve detection of relevant cardiovascular pathologies [1] or unknown cardiac disorders [2], and to allow a good assessment of cardiac anatomy and function [3-5]. Furthermore they also have been shown to ensure a reliable assessment of left ventricular hypertrophy [6] and abdominal aortic aneurysm [7,8]. It has also been suggested that internists may use HHD without formal training in echocardiography and after a limited echo-training period [9-11]. In addition, HHD has demonstrated acceptable accuracy during patient transport [12] and in the context of ultrasound-guided pericardiocentesis and thoracentesis [13]. However, HHD seems to have a narrower diagnostic field when compared with SED in the evaluation of critically ill patients [14,15].

Based on this evidence, HHD may be expected to become an important additional diagnostic tool during outpatients cardiologic consulting or in non-cardiologic hospital sections. Nevertheless, the potential role of HHD in these settings has never been investigated.

## Methods

Eighty-seven consecutive patients (47 males and 40 females, mean age 66.1 ± 15.2 years), who visited our Hospital for cardiologic consulting, have been included in this study. Each participant in the study had one or more referral questions among the following: hypertension, dyspnoea, chest pain, palpitations (Table 1).

**Table 1:** Referral questions in the study population

**Referral question**	**No. of Patients**
**Hypertension**	45 (51.7%)
**Dyspnoea**	34 (39.1%)
**Chest pain**	30 (34.5%)
**Palpitations**	6 (6.9%)
**Pericardial effusion control**	3 (3.4%)

All patients underwent routine physical examination, resting ECG and echocardiographic evaluation using a basic model of HHD (*Opti-Go*, Philips Medical System). The end-diastolic left ventricular (LV) diameter, interventricular septum and posterior wall thicknesses, the size of the aortic root, bulb and ascending segment, and the end-systolic left atrial antero-posterior diameter were measured by B-mode imaging, using the parasternal long-axis view. An estimation of LV ejection fraction and regional wall motion have also been assessed, with computation of the wall motion score index. A gross assessment of aortic, mitral, tricuspidalic and pulmonic valve features (calcifications, abnormal movements) has been performed. The presence and severity of regurgitations has been estimated using colour flow Doppler imaging. The presence of pericardial effusion has been evaluated using multiple views, and subcostal view was used to measure abdominal aortic diameter.

The cardiologist, whenever possible, formulated a diagnosis at the end of the exam. The percentage of subjects for whom the diagnosis was considered satisfactory – i.e., when findings were judged to be reasonably adequate for final diagnostic and therapeutic conclusions and no further diagnostic evaluation was needed – was used to quantify the "conclusiveness" of HHD evaluation. Within 24 hours, all patients underwent a second echocardiographic evaluation, performed by a second cardiologist with similar experience and echocardiographic competence, blinded to the results of the other investigator. The examination has been performed by using a SED (Agilent Technologies, Sonos 5500). The agreement between the first and the second echocardiographic exam has been assessed by controlling the percentage of concordant diagnostic conclusions between the two evaluations.

Data are shown as mean ± SD for continuous variables. The comparison of the examination time between HHD and SED has been assessed using one-way ANOVA. The chi-squared test was used to compare the percentages of conclusiveness between the two ultrasound machines. In the case of expected frequencies ≤ 5, the Fisher's exact test was performed. A P value <0.05 was considered for statistical significance. The statistical analysis was performed using the SPSS (Statistical Package for the Social Sciences, Chicago, Illinois) software.

## Results

### Conclusiveness of HHD and SED

Mean examination time was 6.7 ± 1.5 minutes using HHD and 13.6 ± 2.4 minutes using SED (p < 0.05). The echocardiographic evaluation performed using HHD was considered satisfactory in 74/87 patients, corresponding to 85.1% conclusiveness. After examination using SED, the diagnosis was satisfactory in 83/87 patients, which corresponded to a 95.4% conclusiveness (p = 0.02).

Among the 13 patients in whom HHD examination was not conclusive, 6 had poor acoustic window, 4 showed a colour Doppler pattern suggestive of aortic stenosis, and 3 presented critical conditions. All 4 subjects for whom the SED examination were considered to be satisfactory had inadequate acoustic window.

### Agreement between HHD and SED

**Figure 1:**
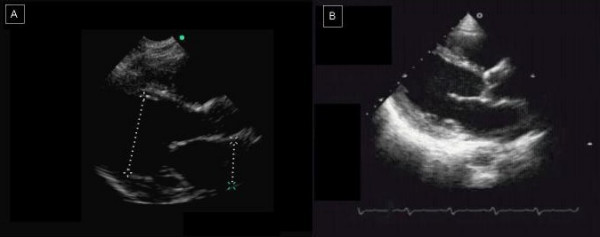
A case of dilatative cardiomyopathy. On the left (A), HHD image in a parasternal long-axis view obtained with HHD. On the right (B), the same patient evaluated with SED.

**Figure 2:**
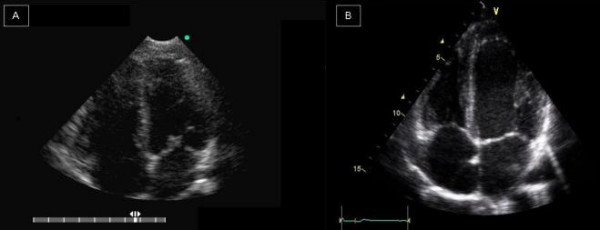
A normal patient. On the left, it’s shown an apical four-chambers view obtained with HHD (A). On the right (B), the same patient evaluated with SED.

Among the 74 patients for whom the examination using the HHD was conclusive, the diagnosis was concordant with that obtained following the examination with the SED in 62 cases (83.8%) (images obtained with HHD and SED both in diseased [Figure 1] and normal [Figure 2] patients). The causes of the 12 diagnostic bias were the following: errors in wall thickness measurements (n = 5); incorrect assessment of mitral regurgitation (n = 3); inaccuracy in evaluation of wall motion abnormalities (n = 3) and in measurement of the diameter of the ascending aorta (n = 1).

### Accuracy according to referral question [Figure 3]

**Figure 3:**
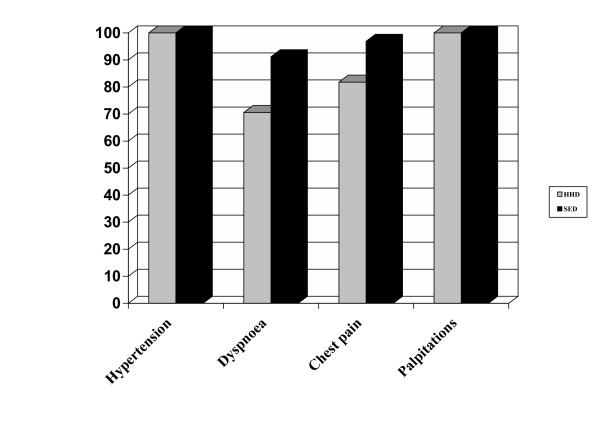
Conclusiveness of echocardiographic examination according to referral question: the  diagnostic accuracy of the HHD seems to be affected by the type of referral question. HHD showed  a good reliability in the examination of patients referred for arterial hypertension or palpitations, but  was less accurate for those who presented dyspnoea or chest pain, as a result of biases in the  measurements of wall thicknesses, and in the assessment of valvular diseases and regional wall  motion.

Examinations performed using either the HHD or the SED were considered satisfactory in all 45 patients referred for arterial hypertension, corresponding to 100% conclusiveness. In 3 of them a diagnostic bias occurred using the HHD, because of incorrect wall thickness measurement. This corresponded to a 93.3% agreement between HHD and SED.

Among 34 patients evaluated for dyspnoea, the examination has been considered conclusive in 27 subjects (70.6%) using the HHD and in 31 (91.2%, p = 0.11) using the SED. The causes for unsatisfactory examinations using the HHD were: poor acoustic window (n = 3 for both HHD and SED), suspected aortic stenosis (n = 2) and critical conditions (n = 2). Among the 27 patients with conclusive examination by the HHD, a diagnostic bias has been found in 4, because of errors in wall thickness measurements (n = 1), incorrect mitral regurgitation staging (n = 2), and missed ascending aorta enlargement diagnosis (n = 1). This yielded a 58.8% agreement between HHD and SED results.

Among the 33 patient with chest pain, a conclusive examination has been obtained in 27 subjects (81.8%) using the HHD and in 32 (96.9%) using the SED (p = 0.30). Three of the six patients with unsatisfactory examination using the HHD had poor acoustic window (the cause of unsatisfactory examination using SED was a poor acoustic window, too), two had aortic stenosis, whereas in one subject, who showed critical conditions, the examination was not conclusive using HHD. Among the 27 patients with conclusive diagnosis following HHD examination, a diagnostic bias was observed in 5 cases: 3 because of inaccuracy in evaluation of wall motion abnormalities, 1 for inaccuracy in mitralic valve assessment and 1 for errors in wall thickness measurements. This leaded to a percentage of agreement between HHD and SED of 72.7%.

Lastly, among 6 patients referred for palpitations, conclusiveness and agreement between the two techniques were 100%.

## Discussion

This is the first study, which evaluates the clinical utility of a basic model of HHD in the context of cardiologic consulting for outpatients or in non-cardiologic hospital sections. The clinical usefulness of the HHD has been reported in previous studies [16-21]. Potential advantages which could result from the use of a basic HHD include brief examination time, simplicity of use, fast availability at the patient's bedside, easy transportability and relatively low cost. Despite limitations due to the lack of M-mode imaging and power, and continuous Doppler analysis, our findings suggest that a basic model of HHD may provide a useful and reliable adjunctive diagnostic tool for cardiologic examination of both outpatients and patients admitted in non-cardiologic sections. The ecocardiographic examination, performed using the HHD, was satisfactory and conclusive in about 85% of subjects, and a good agreement between the diagnosis derived using the HHD and that obtained using the SED was obtained in this subset of patients (83.8%). Thus, a correct diagnosis was made in 71.3% of the total study population. It should also be considered that better-equipped HHD show similar advantages and may ensure further higher diagnostic accuracy in comparison with basic models, thanks to several improvements (M-mode imaging, pulsed and continuous wave Doppler facilities, ECG synchronisation, storage memories, multiple peripheral connections) [22], but they are more expensive. It is also to be expected that such hand-carried ultrasound devices will soon become smaller, simpler to be used and cheaper [23], similar to an "ultrasound stethoscope" [22].

Some examinations resulted "unconclusive" or unsatisfactory due to a poor acoustic window, either for HHD than SED, so this bias represents a limit for both of them.

However, basic HHD should not be considered equivalent to SED. The diagnostic accuracy of the HHD seems to be affected by the type of referral question. The HHD showed a good reliability in the examination of patients referred for arterial hypertension or palpitations -also allowing a rapid screening of left ventricular hypertrophy [6] and of abdominal aortic aneurysm [7] – but was less accurate for those who presented dyspnoea or chest pain. In the subset of subjects with dyspnoea, the percentage of satisfactory examinations tended to be higher – although not significantly – using the SED compared with the HHD. Also, in the subgroups of patients with dyspnoea or chest pain the agreement between the results obtained using the two ultrasound machines was suboptimal, mostly as a result of biases in the measurements of wall thicknesses, and in the assessment of valvular diseases and regional wall motion. These results suggest that the diagnostic information obtained using the HHD should be critically considered in patients with chest pain or dyspnoea.

In this study we evaluated HHD comparing its clinical utility to SED: so, SED was our Gold Standard; the number of patients was also very limited (87 patients examined).

Moreover, we don't know if HHD evaluation combined with ECG and physical examination, *vs *ECG and physical examination only, really allow us to obtain more accurate clinical conclusions.

## Conclusion

HHD real utility is allowing evaluating patients with a gain in terms of time, shortening patients waiting lists, and reducing healthy costs. Moreover, HHD evaluation can help the physician in the choose of the therapy and in the follow-up of the patient.

On these basis, HHD may generally allow a reliable cardiologic basic evaluation of outpatient or subjects admitted to non-cardiologic sections. However, in patients with chest pain and dyspnoea, the use of such devices should be performed with caution, and the diagnostic results should be considered critically.
